# From nystagmus to the air and water caloric tests

**DOI:** 10.1590/S1808-86942012000400022

**Published:** 2015-10-20

**Authors:** Anna Carolina Marques Perrella de Barros, Heloisa Helena Caovilla

**Affiliations:** 1 MSc in Sciences - Graduate Program in Human Communication Disorders - Federal University of São Paulo - Paulista School of Medicine (UNIFESP/EPM) (Speech and Hearing Therapist); 2 Senior Associate Professor of Otology and Neurotology - Federal University of São Paulo - Paulista School of Medicine (UNIFESP/EPM) (Speech and Hearing Therapist)

**Keywords:** caloric tests, labyrinthine fluids, nystagmus, physiologic, vestibular function tests, vestibule, labyrinth

## Abstract

The caloric test is an important tool for the assessment of labyrinthine function.

**Objective**: To compare the nystagmus response in the caloric tests with air at 50°C and 24°C and with water at 44°C and 30°C. Study Design: Randomized crossover clinical trial.

**Materials and Methods**: 40 healthy individuals were submitted to a neurotological evaluation, including caloric tests with air at 50°C and 24°C and water at 44°C and 30°C.

**Results**: Comparing the air and water caloric tests, there were no significant differences among the post-caloric nystagmus slow-phase velocity in relation to the stimulation order, between ears and between the values of unilateral weakness and directional preponderance. The slow-phase velocity values were higher with water (*p* = 0.008, ***p*** < 0.001), and cold stimulation produced stronger responses (*p* < 0.001).

**Conclusion**: Comparing 50°C and 24°C air caloric test and 44°C and 30°C water caloric test, we observed similar slow-phase velocity values for both ears, higher responses in the cold temperature and in the test with water, and similar results of unilateral weakness or directional preponderance for post-caloric nystagmus in both tests.

## INTRODUCTION

The caloric test is usually the most informative part of vestibular evaluation. It is the only procedure which assesses each labyrinth separately, it informs about the affected side and enables characterization of the vestibular lesion intensity^1^, ^2^, despite having large intra and inter-individual variability in its evaluation parameters^2^, ^3^.

Heat stimulation of the labyrinth causes endolymphatic current in the semicircular canals, polarizing or depolarizing the sensorial cells of the ampule crest, triggering the vestibular-ocular reflex. The stimulus generates an ampule current, towards the utricle, exciting the stimulated lateral semicircular canal and causing nystagmus in the same direction of the labyrinth being tested. The cold stimulus results in a current in the opposite direction of the utricle, inhibiting the stimulated semicircular canal and nystagmus in the opposite direction of the assessed labyrinth^2^, ^3^, ^4^, ^5^.

Besides the convection currents, other mechanisms may be involved in evoking caloric responses, such as the mechanical transduction of the cupule, the heat effect on sensorineural structures and neural adaptation, combined in a non-linear fashion^2^.

Under physiological conditions, the vestibular organs are sensitive to stimuli varying between 0.001 Hz and 8 Hz^6^, and the semicircular canals respond more efficiently to angular movements between 1 and 6Hz^7^.

The caloric test enables the assessment of the lateral semicircular canal; defects must be interpreted together with other tests, since they do not necessarily indicate that the labyrinth is involved^8^. This tests provide partial information about the vestibular function, because it causes an endolymphatic current in the corresponding lateral semicircular canal at only 0.003 Hz of the head angular movement^7^.

Many methods have been created in order to assess vestibular function by means of heat stimulation. Initially, water was the only element employed to evoke the nystagmic response^9^. Ether and ethyl chloride^10^ have also been used, and the biphasic caloric test with air has been advocated, in which hot and cold temperatures are quickly alternated during the stimulation, producing a bidirectional nystagmus^11^; the caloric test with continuous thermal change, in which the temperature is regulated during the test and reduced at a constant rate until nystagmus ensues^12^; and one with a silicone balloon in the external acoustic meatus with circulating water, which enables the test to be carried out in ears with perforated ear drums^13^; stimulation could be alternate bithermal, simultaneously bithermal, monothermal and by means of cold tests only^2^.

Air irrigators became commercially available around the 60‘s^14^ and are broadly used today. In Brazil, one air caloric stimulation test equipment used, the Ototermoar, caused a reaction in the labyrinth similar to that with water in healthy individuals and differentiated healthy individuals from patients with vestibular disorders, enabling the conclusion that the air test was technically doable, and could be used in clinical practice^15^.

Air is more comfortable and safer for the test, without contraindications to its use, and it can be used in the assessment of patients with eardrum perforations, patients with external otitis media and mastoidectomy cavities^2^, ^10^, ^16^, ^17^, ^18^. Differently from water, it does not need to be collected during irrigation and the necessary time to reach the warmer temperature is shorter^9^.

The thermal properties of air and water are significantly different. The capacity to maintain the air temperature is lower than that of water^19^, ^20^. The technical demand for the test with air is higher^16^, though. The model and diameter of the irrigator tip, how much is inserted into the external acoustic meatus, the direction and flow of air, the room temperature and the location of the equipment in the testing room may affect the characteristics of the air; thus, the amplitude of post-caloric responses^3^, ^16^, ^17^, ^21^.

The literature discusses the effort of researchers to define the best procedure and to obtain the caloric response with air. The temperatures utilized in the caloric test with hot air varies between 42°C and 51°C and in cold test, they vary between 20°C and 30°C, with air flows between 5 and 13L/min and stimulation time between 60 and 100 seconds^10^, ^14^, ^17^, ^18^, ^20^, ^22^, ^23^.

In the irrigation with water, the temperatures employed are 7°C higher and lower than body temperature; 44°C for the warm test and 30°C for the cold one. In the irrigation with air, one needs gradients of 13°C, corresponding to 50°C, for the hot stimulation and to 24°C for the cold one, in order to evoke a response with amplitude similar to the one obtained by water stimulation^2^, ^16^, ^23^.

The most important parameter for the caloric test quantitative assessment, which can be analyzed thanks to the possibility of recording the response, is the maximum Slow Component Angular Velocity (SCAV), which is proportional to the intensity of the employed stimulus^24^. The hot and cold stimulations with air usually produce nystagmic responses which are similar to the ones produced with water^19^, ^25^, with response reliability similar to that of the test with water^9^, ^19^ and proper test-retest reliability^26^. Nonetheless, there is a report that the absolute SCAV values were more intense in the test with water^17^, ^20^ and showed greater variability in the test with air^22^, ^27^. Different responses in the comparison between hot and cold stimuli are not very important, since the prime goal of the caloric test is to show whether the response to the same hot or cold stimulus is symmetrical in both ears^28^.

The caloric stimulation with air was challenged because of response variability^9^, ^16^, ^27^. The variability of post-caloric responses with air could be explained by the tendency of air temperature balancing out with the room temperature. The use of temperatures farther from the body temperature and the increase in irrigation time and flow may broaden response intensity; the test-retest variations drop when the stimulus intensity and flow are increased, and when the temperature is closer to that of the room^22^. At levels above 10 L/min, the discomfort caused by the acoustic stimulus is higher^22^ and, at levels below 5 L/min, the heat transfer is markedly reduced^23^.

Recent studied on the caloric test with water and air usually approach topics associated with the findings in vestibular disorders. In our country, in caloric tests with air at 42°C and 18°C and with water at 30°C and 44°C in chronic peripheral vestibular disorders, they found a prevalence of cases with SCAV values abnormalities on labyrinthine predominance and directional preponderance^29^.

The diagnostic importance of the caloric test and the scarce information in our literature on the comparison between thermal labyrinthine stimulations with water and with air motivated this study.

The temperatures of 50°C and 24°C of the caloric test with air has been recommended by advanced neurotology centers of numerous countries; and in our country there is no scientific paper comparing the results of this test with the ones from the conventional caloric test with water.

The present study can be very practical for the routine assessment of vestibular function and it also opens horizons for other studies with the caloric test with air in these temperatures, enabling a pertaining comparison with the results from the international literature.

The goal of the present study is to compare the post-caloric nystagmus of the test with air at 50°C and 24°C with that from the test with water at 44°C and 30°C.

## MATERIALS AND METHODS

The present study was carried out after approval by the Ethics in Research Committee of the institution, under protocol number 0054/09. The informed consent form was signed by all the subjects before the study onset.

The study group was made up of 40 volunteers, 25 females and 15 males, with ages between 18 and 40 years, without ear complaints.

As for inclusion criteria, we selected healthy individuals without signs and symptoms of body balance disorder and who were not using medication. We carried out a guided interview to rule out a past of neurotological diseases and use of medication. We also excluded healthy individuals with changes in the vestibular test with spontaneous nystagmus with eyes closed and/or pre-caloric, because of the possible influence of these signs on the caloric test.

After visually examining the external ear canal, to make sure there was no wax or foreign body, the subjects were submitted to vestibular function assessment^4^, made up of static and dynamic balance tests; positional and positioning nystagmus study; and study of spontaneous and semi-spontaneous nystagmus, saccadic movements, pendular tracking, optokinetic nystagmus, decreasing pendular rotational test and caloric test with air at 50°C and 24°C and with water at 44°C and 30°C with digital vector electronystagmography (VENG - Neurograff Eletromedicina Ind. e Com. Ltda. - EPT - Brazil), using the otocalorimeter with air (model OAT 10, adapted for temperatures of 50°C and 24°C, Neurograff Eletromedicina Ind. e Com. Ltda. - EPT - Brazil) and otocalorimeter with water (model OC 214 - Berger). The responses were registered and analyzed in the VecWin 2 (Neurograff Eletromedicina Ind. e Com. Ltda - EPT - Brazil) software. Before data collection, the otocalorimeters were calibrated, with temperature adjustments according to the technical specs from the manufacturers.

The individuals were distributed in two groups in a randomized fashion, according to the caloric test, which was carried out first. Group I was initially submitted to the caloric test with air and Group II was initially submitted to caloric test with water, in order to avoid a possible influence in the order of stimulations, habituation and residual effects of one test on the other^23^.

Before the functional assessment of the vestibular system, the patients were instructed to refrain from smoking, eating chocolate, drinking alcoholic or caffeine loaded beverages in the three days before the assessment. On the day of the test, they were instructed to refrain from using makeup, facial cremes or contact lenses.

In the exam room, the lighting was dimmed, and the room had air conditioning, keeping the room temperature at about 21°C, to avoid interference from air and water thermal variations in the results.

In order to carry out the VENG, the patients had their skins cleaned in order to enable the placement of three active silver chloride leads and one ground lead. The leads were placed in the right external periorbital corner, in the left external periorbital corner and in the frontal midline, using the triangular placement of the leads which enables the identification of nystagmus direction and to check the slow component angular velocity (SCAV) in three recording channels^30^.

The calibration of eye movements was done in the beginning and, when necessary, throughout the test and, systematically before the caloric test to minimize changes to the response amplitude caused by the cornea-retina electrical variation.

During the caloric test with water and air, the subject was kept in the supine position with the head tilted at 30°C^31^. The heat stimulation was carried out in each ear separately, with air at 50°C and 24°C, in a 8L/min flow, for 60 seconds^9^, ^19^, keeping a three-minute interval between stimulations^19^; and with 250 ml of water at 44°C and 30°C, during 40 seconds, with a five-minute interval between stimulations^4^, ^32^. We had a 15-minute interval after the end of the nystagmus response from the last stimulation with caloric test with water or with air and the onset of the first stimulation of the next test with air or water.

Each subject was submitted to eight hot and cold caloric stimulations with air or water, carried out in the following order: hot in the right ear, hot in the left ear, cold in the left ear and cold in the right ear. We used strategies such as conversation, naming and the use of mathematical calculations to stimulate mental activity and to avoid the cortical inhibition of the caloric response. Vertigo, SCAV and direction of the post-caloric nystagmus were analyzed with the eyes closed and eyes open. SCAV was chosen as the parameter to assess response intensity.

The entire post-caloric nystagmus recording was assessed by selecting the most representative stretch of the post-caloric nystagmus record, with the most intense responses in terms of SCAV. The percentage values of the labyrinthine predominance and directional preponderance of the post-caloric nystagmus were calculated using the SCAV values in the four stimulations with air and in the four stimulations with water^33^.

We did a statistical descriptive analysis to characterize the sample. For the quantitative variables we observed minimum and maximum values and calculated the mean and standard deviation values. For the qualitative variables we calculated the absolute and relative frequencies.

We used the chi-square test to assess the homogeneity between the groups I (air-water) and II (water-air) vis-à -vis the age and gender variables.

The *t-Student* test was utilized in the comparative analysis of Groups I (air-water) and II (water-air) vis-à -vis the mean SCAV value of the post-caloric nystagmus.

We used the paired *t-Student test* in the comparative analysis between the right and left ears in the tests with air at 50 and 24°C and with water at 44°C and 30°C, in the comparative analysis by ear between the tests with air, at 50°C and 24°C, and with water at 44°C and 30°C, in the comparative analysis between the tests with air at 50°C and 24°C and with water at 44°C and 30°C in each ear and in the comparative analysis between the relative values of labyrinthine predominance and nystagmus directional preponderance in the tests with air at 50°C and 24°C and with water at 44°C and 30°C.

The statistical analyses were done by the SPSS 15.0 for Windows (*Statistical Package for Social Sciences*, version 15.0, 2006) software; the significance level employed was 5% (α= 0.05). The power of the tests was calculated when there was significant difference between the groups; the values found varied between 87% and 100.0%, indicating that the sample size was enough to obtain more than 80% of power.

## RESULTS

We assessed 40 healthy individuals, 25 (62.5%) females and 15 (37.5%) males, with mean age of 26.45 years (ranging between 18 and 39 years) and standard deviation of 5.62 years. All the individuals reported having vertigo upon caloric stimulation with air and with water.

Table 1 shows the descriptive values of the distribution of the healthy individuals in Groups I (air-water) and II (water-air) and the comparative analysis between the groups as to age and gender. There was no statistically significant difference between the two groups vis-à -vis age and gender.

**Table 1:** Descriptive values of the distribution of healthy individuals in the two study groups and comparative analysis of the age and gender variables.

Groups				
Variable	Category	I (Air-Water)	II (Water-Air)	*p*-value
Age (in years)		24.95 + 5.80	27.95 + 5.13	0.091(1)
Gender	FemaleMale	12 (60.0%) 8 (40.0%)	13 (65.0%) 7 (35.0%)	0.744(2)

(1) *t* test descriptive probability test.

(2) chi-square descriptive probability test.

Table 2 depicts the mean, standard deviation, minimum and maximum values of the slow component angular velocity of the post-caloric nystagmus in the tests with air at 50°C and 24°C and with water at 44°C and 30°C in the right and left ears, and the *p*-value of the comparative statistical analysis, considering the order at which the tests were performed. Regardless of such order, there were no statistically significant differences between the post-caloric nystagmus SCAV values with air and there were no statistically significant differences between the post-caloric nystagmus SCAV with water.

**Table 2:** Post-caloric nystagmus descriptive values in stimulation with air at 50°C and 24°C and with water at 44°C and 30°C and comparative analysis of the results between the groups.

Caloric test	Temperature/Ear	Groups	n	Mean	SD	Minimum	Maximum	*p*-value
With air	50°C/right	I	20	12.8	5.1	4.1	22.4	0.136
		II	20	9.85	4.12	4.8	23.1	
	50°C/left	I	20	11.31	5.59	2.9	22	0.246
		II	20	9.35	4.86	3.5	22.8	
	24°C/right	I	20	12.91	5.35	5.1	28.4	0.689
		II	20	13.55	4.55	7.3	20.7	
	24°C/left	I	20	14.42	5.34	4.4	23.6	0.55
		II	20	13.56	3.42	8.4	21.7	
With water	44°C/right	I	20	13.21	4.39	6.5	24.2	0.423
		II	20	12.11	4.16	5.6	19.2	
	44°C/left	I	20	12.38	4.81	6.2	22.6	0.911
		II	20	12.56	5.29	6.1	25.4	
	30°C/right	I	20	20.9	7.97	6.6	33.9	0.62
		II	20	22.33	10	9	48.9	
	30°C/left	I	20	22.38	10.08	7.1	46.8	0.795

SD: standard deviation; Student *t* test; Significance level α= 0.05; * Significant values.

Table 3 depicts the mean, standard deviation, minimum and maximum SCAV values and labyrinthine predominance and directional preponderance of post-caloric nystagmus upon stimulation with air at 50°C and 24°C and with water at 44°C and 30°C, and the comparative statistical analysis of the results between the ears, the temperatures and the tests. There was no statistically significant difference upon comparison of the SCAV values between the ears in the caloric tests with air and water. There was a statistically significant difference (*p* < 0.001) between the SCAV values upon comparison of the test with hot air with that of cold air and upon comparing the hot water with the cold water tests in each ear; the cold temperature evoked more intense responses than the hot temperature in the tests with air and water. There was a statistically significant difference (*p* = 0.008; *p* < 0.001) between the caloric test with air in relation to the test with water in all the stimulations; the SCAV values were higher in the stimulations with water than air. There were no statistically significant differences between the values of labyrinthine predominance (*p* = 0.761) and directional preponderance (*p* = 0.391) upon comparison of the results from the tests with water and air.

**Table 3:** Post-caloric nystagmus descriptive values at stimulation with air at 50°C and 24°C and with water at 44°C and 30°C and comparative statistical analysis of the results between the ears, the temperatures and the tests.

Caloric test	Temperature/Ear	n	Mean	Standard deviation	Minimum	Maximum
With air	50°C/right	40	10.97	4.71	4.1	23.1
	50°C/left	40	10.33	5.27	2.9	22.8
	24°C/right	40	13.23	4.92	5.1	28.4
	24°C/left	40	13.99	4.45	4.4	23.6
	LP	40	7.35	4.81	0	18.45
	DPN	40	7.22	7.29	0	32.02
With water	44°C/right	40	12.66	4.25	5.6	24.2
	44°C/left	40	12.47	4.99	6.1	25.4
	30°C/right	40	21.62	8.95	6.6	48.9
	30°C/left	40	22.79	9.67	7.1	46.8
	LP	40	7	5.38	0.46	23.1
	DPN	40	6.06	5.39	0	18.63

LP: labyrinthine predominance; DPN: directional preponderance; *p*-value:

Air 50°C/right vs. Air 50°C/left: *p* = 0.132;

Air 24°C/right vs. Air 24°C/left: *p* = 0.211;

Water 44°C/right vs. Water 44°C/left: *p* = 0.654;

Water 30°C/right vs. Water 30°C/left: *p* = 0.218;

Air 50°C/right vs. Air 24°C/right: < 0.001*;

Air 50°C/left vs. Air 24°C/left: < 0.001*;

Water 44°C/right vs. Water 30°C/right: < 0.001*;

Water 44°C/left vs. Water 30°C/left: < 0.001*;

Air 50°C/right vs. Water 44°C/right: *p* = 0.008*;

Air 50°C/left vs. Water 44°C/left: *p* < 0.001*;

Air 24°C/right vs. Water 30°C/right: < 0.001*;

Air 24°C/left vs. Water 30°C/left: < 0.001*;

Air LP vs. Water LP: 0.761;

Air DPN vs. Water DPN: 0.391;

Student *t* Test; Significance level α= 0.05;

* Significant values.

## DISCUSSION

Comparing the results from the air-water (Group I) and water-air (Group II) groups, as to the order in which the tests were carried out, there was no statistically significant difference between the post-caloric nystagmus SCAV with air and between the post-caloric SCAV values with water, regardless of which test preceded the other, ruling out a possible effect of the order of post-caloric responses. Similarly to our findings, in another study, there was no evidence of post-caloric nystagmus physiological adaptation in normal individuals, because the order in which the stimulations were done also did not impact response amplitude^34^.

There were no statistically significant differences when we compared the responses from the right ear with those from the left ear in the stimulations at 50°C and 24°C in the caloric test with air and at 44°C and 30°C with water, similarly to what has been reported by other authors^17^, ^23^.

We noticed that SCAV values were higher with water stimulation when compared to those with air, for both ears. Other authors have also reported higher evoked post-caloric responses with water than air^14^, ^20^, ^26^ or similar responses^10^, ^17^, ^19^, ^23^.

There was a statistically significant difference in the comparative analysis of the hot stimulations (50°C with air and 44°C with water) and the stimulations with cold temperatures (24°C with air and 30°C with water) in each ear. The cold temperature caused more intense responses than the hot one in both tests: with air and with water. Similarly to our findings, there are reports of higher responses in the cold temperature than in the hot one using water^26^, ^28^, ^35^ and air^26^. Notwithstanding, other studies have shown that the hot stimulus caused more intense responses than its cold counterpart in the caloric test with water^9^, ^14^ and with air^9^, ^11^, or that similar results were obtained with both temperatures in the tests with air^14^, ^17^, ^19^, ^23^ and with water^17^, ^19^, ^23^.

There was no significant difference between the relative values of labyrinthine predominance and nystagmus directional preponderance in the caloric tests with air and water, similarly to what was found in other studies^14^, ^23^. The classical indicator of peripheral vestibular lesions is the comparison of the responses from both ears in the bithermal caloric test, by using the labyrinthine predominance formula^22^. Using air as a caloric stimulus does not impact the clinical measure of labyrinthine predominance^27^.

The use of different configurations of flow, duration and temperature of the thermal stimulus in the assessment of the vestibular function^10^, ^14^, ^17^, ^18^, ^20^, ^22^, ^23^, and the use of different protocols to perform the caloric test with water and air, make it difficult to compare and to integrate the findings from many research centers, thus being necessary that each laboratory set its own reference values^36^.

The differences found in the post-caloric responses upon comparison with other studies in healthy individuals may be justified by the different thermal properties of water and air as vectors of labyrinthine stimulation. Different responses are expected when using water, since it has a higher specific heat than air and it is capable of keeping the temperature in the external ear canal for longer; air satisfactorily replaced water in 95% of the clinical cases seen in a hospital; the remaining 5% had lower labyrinthine function, confirmed with the use of a more intense stimulus: ice water^19^.

Considering the equivalence between the relative values of labyrinthine predominance and post-caloric nystagmus directional preponderance in the test with air and water in the healthy individuals we assessed, and that such parameters are fundamental to identify vestibular dysfunctions, we can state that both tests are useful in neurotological routine testing.

## CONCLUSION

Comparing the tests with air at 50°C and 24°C and with water at 44°C and 30°C, we found two similar values for the slow component angular velocity in both ears to be higher with cold temperature and in the test with water, and similar results of labyrinthine predominance and post-caloric nystagmus directional preponderance in both tests.
